# Society Meetings

**Published:** 1901-05

**Authors:** 


### SOCIETY MEETINGS.

THE Buffalo Academy of Medicine held meetings during the months of March and April, 1901, as follows:

*Section on Obstetrics.*—Tuesday evening, March 26th. Program: Ovarian hernia, Stephen Y. Howell; Pelvic disease and its relation to insanity, Lawrence G. Hanley.

Tuesday evening, April 23d. Program: The operative treatment of prolapsus uteri, M. D. Mann; The correction of face presentations, John V. Woodruff.

*Section on Surgery.*—Tuesday evening, April 2d. Program: Diagnosis and treatment of contusions of the abdomen, Charles L. Scudder, Boston, Mass.; discussion opened by Roswell Park.

*Section on Medicine.*—Tuesday evening, April 9th. Program: How are we educating our children? A. W. Bayliss; discussed by Henry P. Emerson, Esq., superintendent of public instruction; P. W. VanPeyma, of the board of school examiners; Henry R. Hopkins, and Mr. Adolph Miers, instructor of gymnastics of the Buffalo gymnasium.

*Section on Ophthalmology, Otology and Laryngology.*—Monday evening, April 15th. Program: (1) A point in physiological optics; (2) A new form of artificial eye, Lucien Howe. A case of primary tuberculosis of the eye, Edmund E. Blaauw.

*Section on Pathology.*—Tuesday evening, April 16th. Program: Report of a case of retroperitoneal sarcoma, Chas. E. Congdon; The pathology of Asthma, Geo. N. Jack.

---

THE National Confederation of State Medical Examining and Licensing Boards will hold its annual meeting at St. Paul, Minn., June 3, 1901, under the presidency of Dr. J. N. McCormack, of Bowling Green, Ky. The committee on Interstate Reciprocity and Uniform Medical Legislation will present its report and the meeting will be one of great interest and importance. The present officers are: President, J. N. McCormack, Bowling Green; vice-presidents, N. R. Coleman, Columbus; James A. Egan, Springfield, Ill.; secretary-treasurer, A. Walter Suiter, Herkimer, N.Y.; executive council —Chairman, William S. Foster, Pittsburg; Joseph M. Mathews, Louisville; William A. Spurgeon, Muncie, Ind; William Warren Potter, Buffalo; Augustus Korndoerfer, Philadelphia. Committee on interstate reciprocity and uniform medical legislation– -Chairman, William A. Spurgeon, Muncie, Ind.; Gardner T. Swarts, Providence; secretary, Emil Amberg, 32 Adams Ave., W., Detroit; Augustus Korndoerfer, Philadelphia; Edwin B. Harvey, Boston.

---

THE American Medical Association will hold its forty-second annual meeting at St. Paul, Minn., Tuesday, Wednesday, Thursday and Friday, June 4,5,6 and 7,1901, under the presidency of Dr. Charles A. L. Reed, of Cincinnati. A large and enthusiastic meeting is anticipated and an elaborate program is in course of preparation.

---

THE American Academy of Medicine will hold its twenty-sixth annual meeting at the Hotel Aberdeen, St. Paul, Minn., on Saturday, June 1, 1901, executive session at 11 a.m., open session beginning at 12 m, and continuing through Monday, June 5, under the presidency of Dr. S. D. Risley, of Philadelphia. The principal features of the meeting will be a symposium on Institutionalism and another on Reciprocity in Medical Licensure. The president's address will be delivered on Saturday evening, June 1, and the annual social session will be held Monday evening, June 3. Members of the profession are always welcomed to the open sessions of the academy. Dr. Charles McIntire, of Easton, Pa., is the secretary.

---

THE American Proctologic Society will hold its third annual meeting at the Hotel Aberdeen, St. Paul, Minn., Tuesday and Wednesday, June 4 and 5, 1901. The following are the officers: President, James P. Tuttle, New York; vice-president, Thomas Charles Martin, Cleveland; sec.-treas., William M. Beach, Pittsburg. Executive Council—Samuel T. Earle, Jr., Baltimore; A Bennett Cooke, Nashville; J. Rawson Pennington, Chicago.

---

THE American Pediatric Society will hold its annual meeting at the International Hotel, Niagara Falls, Monday, Tuesday and Wednesday, May 27, 28 and 29, 1901, under the presidency of Dr. W. T. Booker, of Baltimore. The sessions of the society will be held from 9 a.m. to 12 m., and from 2 to 4 p.m. each day. Papers of high scientific interest are to be read. The profession of Buffalo, Canada and Western New York are most cordially invited to attend. Dr. Samuel S. Adams, of Washington, is the secretary and Dr. Irving M. Snow, of Buffalo, is chairman of the local committee of arrangements.

The Niagara Falls Academy of Medicine held its regular meeting on Monday evening, April 1, 1901, at the office of Dr. N. W. Price. Dr. J. S. Dorian, read a paper on Vaccination.

---

At the 95th annual session of the Medical Society of the State of New York, held at Albany, January 29, 30, 31, 1901, it was moved and unanimously adopted that in order to increase the facilities for becoming permanent members of the society, each county society should be allowed to send five times the number of delegates it had formerly sent to the state society.

These delegates are elected for a term of three years and are eligible for permanent membership if they register twice during that time. This will make the number of delegates from the county societies to the state society 750 in all, or one delegate for every eight or nine members of county societies, without increase or expense to the county societies.

It was further agreed to respond to a widely expressed desire that the society hold a semiannual meeting in the City of New York in the early autumn to be devoted entirely to scientific work and social intercourse.

The officers of the society announce that they have engaged the New York Academy of Medicine for this purpose, where a meeting of two days duration will be held, October 15, 16, 1901.

Members wishing to read papers are requested to communicate with Dr. Nathan Jacobson, 430 South Salina Street, Syracuse; and information of any other nature can be obtained from Dr. Frederic C. Curtis, secretary, 17 Washington Avenue, Albany, or from Dr. Frank Van Fleet, associate secretary, 63 E. 79th Street, New York City.

It is further announced that the society will tender a reception to its members, delegates, and guests on the evening of October 15, at Delmonico's. Tickets of admission to this reception will be furnished without cost to all who register at the semiannual meeting, as well as to the society's guests

---

The Western Ophthalmic and Oto-Laryngologic Association held its sixth annual meeting at Cincinnati, O., April 11 and 12, 1901. The following officers were elected: President, C. R. Holmes, Cincinnati, O.; 1st vice-president, W. L. Dayton, Lincoln, Neb.; 2d vice-president, J. O. Stillson, Indianapolis, Ind.; 3d vice-president, H. W. Loeb, St. Louis, Mo.; treasurer, O. J. Stein, 100 State Street,

Chicago, Ill.; secretary, Wm. L. Ballenger, 100 State Street, Chicago, Ill. Forty new members were elected.

The next meeting will be held in Chicago, April 10, 11 and 12, 1902.

